# Key accelerometry measures for understanding walking sway during dual-task exercises

**DOI:** 10.1016/j.heliyon.2025.e42160

**Published:** 2025-01-24

**Authors:** Abdulaziz A. Alkathiry

**Affiliations:** Department of Physical Therapy and Health Rehabilitation, College of Applied Medical Sciences, Majmaah University, Majmaah, Saudi Arabia

## Abstract

**Aim:**

This study aimed to identify optimal methods of acceleration analysis to accurately detect dual-task-related changes in walking sway.

**Methods:**

Twenty-six healthy adults participated in this study, undergoing various cognitive dual-task conditions while walking. Accelerometers were attached to the lower back to record center-of-mass (COM) acceleration in anterior-posterior (AP) and mediolateral (ML) directions. Data analysis involved multiple computation methods applied to the acceleration data, with comparisons made using different frequency cutoffs and mean referencing.

**Results:**

Analysis revealed significant effects of dual-tasking on walking sway, particularly in AP and combined directions. A 3.5Hz low-pass filter and mean referencing were found effective in capturing these changes. Computation methods, such as root mean square (which quantifies the variability of COM acceleration) and normalized path length (which measures the distance traveled by the COM over time), showed sensitivity to detect changes in sway amplitude during dual-task conditions.

**Conclusion:**

The findings highlight the importance of considering both AP and ML sway in dual-task assessments. Furthermore, the choice of computation method, frequency cutoff, and mean referencing impacted the sensitivity to detect changes in walking sway during dual-tasking. These findings suggest that a 3.5Hz low-pass filter with mean referencing can enhance the sensitivity of dual-task assessments, which could be valuable for clinical balance evaluations or rehabilitation monitoring.

## Highlights

• Dual-task paradigms provide a comprehensive assessment of postural control and balance during challenging activities.
• The effects of dual tasks on sway differ based on direction and filter settings.
• The choice of specific sway parameters and data processing methods can impact the detection of dual-task effects.

## 1 Introduction

Balance and postural control are essential for maintaining an upright posture that enables individuals to walk and function [1]. The normal human body maintains postural control through the processing of visual, proprioceptive, and vestibular input by the brain and the integration of muscle output [2]. Many health conditions, such as neurological disorders, orthopedic injuries, and normal aging, impair balance and postural control [[3], [4], [5]].

In isolation, the balance test may not reveal subtle impairments in postural control and balance deficits. However, when balance and postural control are tested in a dual-task paradigm, the additional cognitive demands of the dual-task can elicit hidden impairments [6]. Dual-tasks assessments provide a more comprehensive evaluation of an individual's postural control, balance, and cognitive function by testing their ability to divide their attention and coordinate motor and cognitive tasks [7].

Dual-task paradigms have been used to increase task complexity to elicit subtle balance impairments that otherwise can be undetected. Dual-task paradigms can range from laboratory-based to clinically-based. The relative ease of use of clinical dual-task can make them well suited to be incorporated into rehabilitation programs [8].

Dual-task exercises are rehabilitation technique that involves performing two tasks simultaneously [9]. Dual-task training aims to improve an individual's ability to perform activities of daily living by requiring the individual to simultaneously perform a primary physical task, such as walking or standing, and a secondary cognitive task, such as counting backward. This type of training is utilized to improve cognitive-motor integration and the individual's ability to carry out daily tasks [10]. Dual-task training is commonly used in older adults and people with neurological conditions such as Parkinson's disease, stroke, or traumatic brain injury, as they are at high risk of falling due to impaired balance [11,12]. Research has shown that dual-task training can lead to improvements in balance, gait, and cognitive function, and may also lead to improved functional abilities, such as the ability to climb stairs or walk on uneven surfaces [12,13].

Body sway has been used as an indicator of balance [14]. Body sway refers to conscious and unconscious movements made to maintain balance. It is typically measured by tracking the center of mass or center of pressure as a person stands or walks. Sway can be measured by various tools such as force plates, motion capture systems, or accelerometers. With the recent interest in the measurement of standing and walking balance, the usage of wearable inertial sensors have surpassed traditional methods of measuring postural sway. These sensors have practical applications, including fall risk assessments, rehabilitation progress monitoring, and athletic training [[15], [16], [17]].

Wearable inertial sensors, such as accelerometers, are becoming increasingly popular in recent years due to advances in technology, which have led to smaller, cheaper, and more accurate sensors [18]. Accelerometers are relatively inexpensive devices that can be worn on the body and used to measure acceleration. Accelerometers can be used in a variety of applications, such as tracking movement during physical activity and measuring posture and balance, which also help to measure balance in individual people with balance disorders [19].

Most accelerometers provide acceleration information of a single time point in 3 directions the anterior-posterior, the medial-lateral, and the vertical direction. Multiple methods of analysis of acceleration data have been proposed and used in the literature to calculate sway parameters [20]. Some of the most common sway parameters include root mean square (RMS), normalized path length (NPL), mean absolute acceleration (MAA), mean frequency of acceleration (MFA), and Peak to Peak acceleration (P2P) [[21], [22], [23], [24]]. It is worth noticing that these parameters are not specific to analyzing sway during dual-task paradigms.

Each of the sway parameters can be performed on acceleration data in the AP and the ML directions and most of them can be performed on the combination of these directions. Furthermore, acceleration data are filtered using different cutoff frequency filters. The filtered acceleration data can be used in its raw form or be standardized before analysis.

This study addresses the lack of consensus on the most sensitive and reliable computational methods for analyzing changes in walking sway induced by dual-tasking [[25], [26], [27], [28]]. While previous research has explored the general utility of accelerometers, this study focuses on comparing specific analysis techniques to determine their effectiveness in detecting subtle sway changes. By identifying the optimal parameters and methods of data analysis, this research aims to enhance the utility of accelerometer-based assessments in clinical and rehabilitation contexts. Walking at a self-selected speed was chosen over static balance tests to better mimic real-world [29,30]. Unlike walking at fixed speeds on a treadmill, self-selected walking allows for natural variations and better reflects individual adaptations to dual-task challenges [29].

The study compared various methods of acceleration analysis to detect changes in walking sway during a dual-task exercise paradigm. It aimed to identify the most sensitive methods for detecting subtle changes in walking sway during dual-task exercises, thereby enhancing balance assessment in clinical and rehabilitation settings. The findings contribute to the development of evidence-based tools for evaluating balance.

## 2 Methods

This research was carried out with the participation of twenty-six adults, both male and female, who were healthy. Adult males and females between the ages of 18 and 60 who were able to walk without walking aids were considered eligible for participation in the study. This study excluded participants who had any neurological deficiencies or injuries to their lower limbs.

All methods were carried out in accordance with relevant guidelines and regulation. All the participants provided their consent to participate and to publish the results of this study. The datasets used and/or analyzed during the current study are available from the corresponding author on reasonable request.

### 2.1 Dual tasks

The dual tasks were a combination of a motor task (which was a 15 s walking at the individual's comfortable speed) and one of five different cognitive tasks. The cognitive tasks were serial subtractions by 7's (DT1), naming words that start with a certain letter (DT2), reverse reciting of the months (DT3), normal conversation (DT4), and reverse spelling (DT5). Before starting the dual task tests the investigator explained the cognitive tasks to the participants. Tasks were performed in a random order and participants were not instructed to focus more on the motor task or the cognitive task. The various cognitive tasks were chosen to introduce varying levels of cognitive demand, which are known to influence postural control differently. Tasks such as serial subtraction or reverse spelling are cognitively demanding and may elicit greater changes in postural control compared to less demanding tasks like normal conversation. Including a range of tasks allowed the study to examine the robustness of the computational methods across different dual-task intensities.

### 2.2 Data analysis

A smartphone-based accelerometer was attached to the participant's lower back at the level of the umbilicus. The accelerometer recorded the participant's center-of-mass (COM) acceleration in the anterior-posterior (AP) and the mediolateral (ML) directions while walking during each of the 5 dual-tasks. The acceleration data were sampled at a rate of 100 Hz. The middle 10 s of acceleration data were used to avoid any effects of walking acceleration and deceleration on the data. The measurement unit for acceleration was the gravitational acceleration constant G (1 G = 9.81 m/s^2^). The accelerometer was calibrated before each session of data collection by flipping the accelerometer to read the gravitational acceleration constant in the positive and the negative directions (9.8 m/s^2^ and -9.8 m/s^2^, respectively) as well as by placing the accelerometer horizontally to show a reading of (0 m/s^2^). Calibrations were performed in the AP and ML directions.

To ensure consistency across participants, the accelerometer was secured using a standardized mounting harness designed to maintain the device perpendicular to the ground. The harness position was verified visually by the investigator before each trial to ensure alignment with the AP and ML axes.

**Fig. 1:**
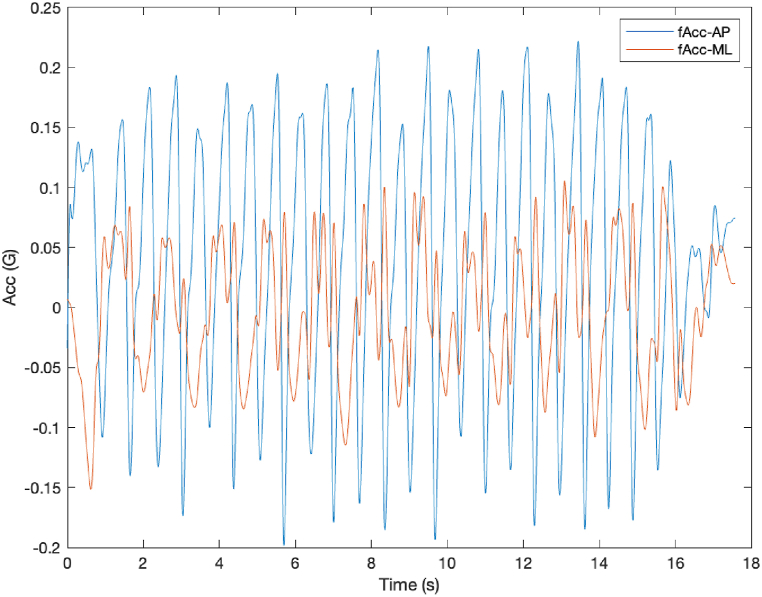
Acceleration data for participants center of mass while walking plotted against time (seconds). G: gravitational acceleration = 9.81 m/s^2^; fAcc: filtered acceleration (2 Hz).

Data quality was assessed by plotting and visually inspecting the acceleration signals. The visual inspection ensured the data followed expected patterns in the AP and ML directions, and any inconsistencies (e.g., sudden spikes or misalignments) were flagged for reevaluation of sensor positioning (Fig. 1).

Raw acceleration data (Acc_raw_), in the AP and ML directions, were analyzed using a custom MATLAB program (Mathworks, Inc., Natick, Massachusetts). The data were processed using two low-pass filters with frequency cutoffs of 2 Hz and 3.5 Hz, generating 2 sets of filtered acceleration data (Acc) (Codes 1 and 2). These 2 sets of Acc were then mean-referenced (Acc_x_) (Equation (1)). The combinations of filters (2 Hz and 3.5 Hz) and mean-referencing (Acc and Acc_x_) resulted in 4 distinct sets of acceleration data (2Hz-Acc, 2Hz-Acc_x_, 3.5Hz-Acc, and 3.5Hz-Acc_x_)

Additionally, the AP and ML directions were combined to generate two-dimensional acceleration (2D) (Equation (2)) using each of the 4 sets of acceleration data.

Five computational methods were applied (RMS, NPL, MAA, MFA, and P2P) (Equations (3), (4), (5), (6), (7), (8))) for the AP, ML, and 2D data to each of the 4 sets of acceleration data. However, P2P computation method was not applicable to acceleration data in the 2D direction. In total, 56 sway measures were computed for each of the 5 dual-task conditions.

**Code 1: low-pass filter with frequency cutoff of 2 Hz**
  Norder=2;
  Wp=(2)/(100∗0.5);
  [b,a]=butter(Norder,Wp);
  $$\text{Acc}=\text{filtfilt}\left(b,a,{\text{Acc}}_{\text{raw}}\right)\text{;}$$

**Code 2: low-pass filter with frequency cutoff of 3.5 Hz**
  Norder=2;
  Wp=(3.5)/(100∗0.5);
  [b,a]=butter(Norder,Wp);
  $$\text{Acc}=\text{filtfilt}\left(b,a,{\text{Acc}}_{\text{raw}}\right)\text{;}$$

**Equation 1: Mean referenced acceleration**
  (1) $${Acc}_{X}\left[n\right]=Acc\left[n\right]-\bar{Acc}$$
  (1) Acc‾=1N∑Acc[n]
  Acc: acceleration including AP.Acc, and ML.Acc each performed separately.
  Acc [n]: acceleration sample.
  $\bar{Acc}$: average acceleration
  ${Acc}_{X}\left[n\right]$: acceleration sample referenced to the mean

**Equation 2: Combining AP and ML directions**
  (2) $$2D.Acc\left[n\right]=\sqrt{{AP.Acc\left[n\right]}^{2}+{ML.Acc\left[n\right]}^{2}}$$
  Acc: include the 2Hz and the 3.5Hz filters and the Acc and Acc_X_ data.
  2D.Acc[n]: acceleration sample in the combined AP and ML directions
  AP.Acc[n]: acceleration sample in the anterior posterior direction
  ML.Acc[n]: acceleration sample in the mediolateral direction

**Equation 3: Root mean square (RMS)**
  (3) RMS=1N∑Acc[n]2
  Acc: acceleration including AP.Acc, AP.Acc_x_, ML.Acc, ML.Acc_x_, 2D.Acc, and 2D.Acc_x_ each performed separately.
  RMS: Root mean square of acceleration.
  *N*: number of acceleration samples.
  Acc[n]: acceleration sample

**Equation 4: Normalized path length (NPL)**
  (4) $$NPL=\left[\sum_{n=1}^{N-1}\left|Acc\left[n+1\right]-Acc\left[n\right]\right|\right]/\;T$$
  Acc: acceleration including AP.Acc, AP.Acc_x_, ML.Acc, ML.Acc_x_, 2D.Acc, and 2D.Acc_x_ each performed separately.
  NPL: normalized path length of acceleration.
  *N*: number of acceleration samples.
  Acc [n]: acceleration sample.
  Acc [n+1]: the following acceleration sample.
  T: time in seconds.

**Equation 5: Mean absolute acceleration (MAA)**
  (5) MAA=1N∑|Acc[n]|
  Acc: acceleration including AP.Acc, AP.Acc_x_, ML.Acc, ML.Acc_x_, 2D.Acc, and 2D.Acc_x_ each performed separately.
  MAA: mean absolute acceleration.
  *N*: number of acceleration samples.
  Acc [n]: individual acceleration sample.

**Equation 6: Mean frequency of acceleration in AP or ML direction (MFA)**
  (6) MFA=∑n=1N−1|Acc[n+1]−Acc[n]|42(1N∑|Acc[n]|)∗T
  Acc: acceleration including AP.Acc, AP.Acc_x_, ML.Acc, and ML.Acc_x_ each performed separately.
  MFA: Mean frequency of acceleration.
  *N*: number of acceleration samples.
  Acc [n]: individual acceleration sample.
  Acc [n+1]: the following individual acceleration sample on the time series.
  T: time in seconds.

**Equation 7: Mean frequency of acceleration (MFA) in the combined directions (2D)**
  (7) MFA2D.Acc=∑n=1N−1|2D.Acc[n+1]−2D.Acc[n]|2π(1N∑|2D.Acc[n]|)∗T
  2D.Acc: includes 2D.Acc and 2D.Acc_X_ each performed separately.
  MFA: Mean frequency of acceleration.
  *N*: number of acceleration samples.
  2D.Acc [n]: individual acceleration sample.
  2D.Acc [n+1]: the following individual acceleration sample on the time series.
  T: time in seconds.

**Equation 8: Peak to Peak acceleration**
  (8) P2P=Max.Acc−Min.Acc
  Acc: includes AP.Acc, AP.Acc_x_, ML.Acc, and ML.Acc_x_ each performed separately.
  P2P: Peak to Peak acceleration.
  Max.Acc: the maximum acceleration value.
  Min.Acc: the minimum acceleration value.

### 2.3 Statistical analysis

The statistical analysis was conducted using IBM SPSS Statistics for Macintosh (version 28; IBM Corp, Armonk, NY) with a significance level of α = 0.05. The distribution of the acceleration data was tested using Shapiro-Wilk test to assess normality.

Descriptive statistics, including means, standard deviations, and ranges, were calculated for all sway parameters. Four one-way repeated measures multivariate analyses of variance (MANOVA) were performed to compare the five dual-task conditions using each of the 4 sets of acceleration data. Each MANOVA included multiple dependent variables representing sway parameters (RMS, NPL, P2P, MFA, and MAA) and directions (AP, ML, and 2D). The assumption of sphericity was tested using Mauchly's test, and when this assumption was violated, the Greenhouse-Geisser correction was applied. Pairwise comparisons with Bonferroni adjustments were conducted to identify specific differences between tasks.

## 3 Results

**Fig. 2:**
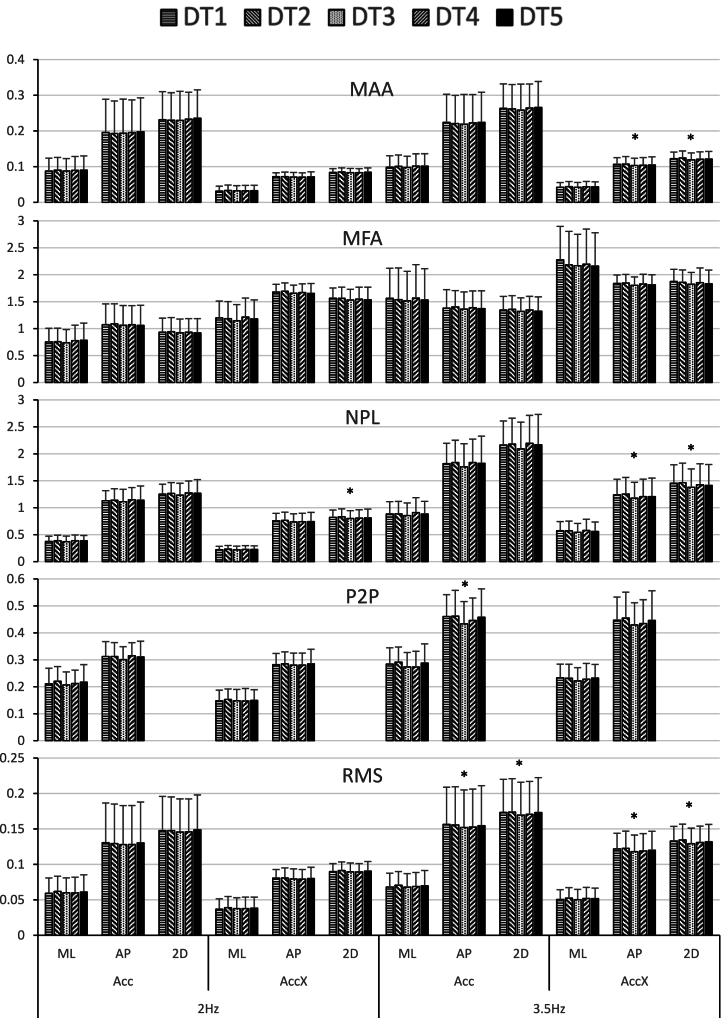
Mean and SD of acceleration data for participants center of mass while walking. RMS: root mean square; P2P: Peak to Peak acceleration; NPL: normalized path length; MFA: mean frequency of acceleration; MAA: mean absolute acceleration; ML: mediolateral direction; AP: anterior-posterior direction; 2D: combined direction; Acc: filtered acceleration; Acc_X_: mean referenced filtered acceleration; 2Hz: 2 Hz cutoff frequency of a low pass filter; 3.5Hz: 3.5 Hz cutoff frequency of a low pass filter; DT1: serial subtractions by 7's; DT2: naming words that start with a certain letter; DT3: reverse reciting of the months; DT4: normal conversation; DT5: reverse spelling; ∗: Significant dual-task effect (p < 0.05).

Twenty-six healthy male and female adults (mean age = 29, SD = 8 years) participated in the study. Fig. 2 provides a visual summary of the results of their sway. The means and standard deviations for sway are presented in (Table 1).

**Table 1:** Descriptive Statistics for Sway Measures Across Dual-Task Conditions.

Direction				AP					ML					2D				
Filter	Normalization	Measures		Dual Tasks					Dual Tasks					Dual Tasks				
				DT1	DT2	DT3	DT4	DT5	DT1	DT2	DT3	DT4	DT5	DT1	DT2	DT3	DT4	DT5
2Hz	Acc	MAA	M (SD)	0.196 (0.093)	0.193 (0.091)	0.194 (0.095)	0.196 (0.091)	0.198 (0.095)	0.088 (0.036)	0.090 (0.036)	0.088 (0.035)	0.090 (0.039)	0.090 (0.040)	0.231 (0.079)	0.230 (0.077)	0.230 (0.081)	0.233 (0.075)	0.236 (0.079)
		MFA	M (SD)	1.075 (0.384)	1.088 (0.373)	1.062 (0.367)	1.072 (0.354)	1.060 (0.372)	0.753 (0.253)	0.757 (0.251)	0.738 (0.245)	0.777 (0.287)	0.784 (0.318)	0.933 (0.261)	0.945 (0.262)	0.923 (0.256)	0.933 (0.253)	0.921 (0.264)
		NPL	M (SD)	1.131 (0.185)	1.139 (0.214)	1.112 (0.229)	1.148 (0.226)	1.139 (0.264)	0.374 (0.097)	0.387 (0.103)	0.370 (0.103)	0.388 (0.106)	0.387 (0.098)	1.251 (0.184)	1.267 (0.200)	1.233 (0.220)	1.274 (0.221)	1.268 (0.254)
		P2P	M (SD)	0.312 (0.055)	0.313 (0.051)	0.301 (0.048)	0.315 (0.048)	0.311 (0.058)	0.211 (0.058)	0.220 (0.055)	0.207 (0.048)	0.212 (0.050)	0.217 (0.065)					
		RMS	M (SD)	0.131 (0.056)	0.129 (0.056)	0.128 (0.055)	0.128 (0.055)	0.130 (0.058)	0.059 (0.022)	0.062 (0.021)	0.060 (0.021)	0.060 (0.022)	0.061 (0.024)	0.148 (0.048)	0.148 (0.047)	0.146 (0.047)	0.146 (0.046)	0.149 (0.049)
	Acc_X_	MAA	M (SD)	0.072 (0.011)	0.072 (0.013)	0.071 (0.013)	0.070 (0.012)	0.071 (0.014)	0.031 (0.014)	0.033 (0.015)	0.032 (0.014)	0.032 (0.015)	0.033 (0.015)	0.084 (0.011)	0.085 (0.011)	0.083 (0.011)	0.083 (0.011)	0.084 (0.012)
		MFA	M (SD)	1.679 (0.143)	1.694 (0.154)	1.656 (0.151)	1.670 (0.160)	1.655 (0.183)	1.198 (0.313)	1.185 (0.317)	1.146 (0.301)	1.215 (0.351)	1.182 (0.351)	1.564 (0.19)	1.562 (0.207)	1.530 (0.199)	1.548 (0.221)	1.534 (0.234)
		NPL	M (SD)	0.758 (0.137)	0.765 (0.152)	0.738 (0.150)	0.741 (0.156)	0.743 (0.170)	0.222 (0.062)	0.232 (0.068)	0.220 (0.068)	0.228 (0.069)	0.226 (0.067)	**0.821 (0.136)**	**0.832**^**#**^**(0.147)**	**0.800**^**#**^**(0.146)**	**0.808 (0.155)**	**0.810 (0.164)**
		P2P	M (SD)	0.282 (0.042)	0.285 (0.045)	0.280 (0.045)	0.280 (0.045)	0.285 (0.054)	0.148 (0.039)	0.153 (0.039)	0.148 (0.042)	0.148 (0.046)	0.150 (0.040)					
		RMS	M (SD)	0.081 (0.012)	0.081 (0.014)	0.080 (0.014)	0.079 (0.014)	0.080 (0.016)	0.037 (0.015)	0.039 (0.016)	0.038 (0.015)	0.038 (0.016)	0.038 (0.015)	0.090 (0.011)	0.091 (0.012)	0.090 (0.012)	0.089 (0.012)	0.090 (0.014)
3.5Hz	Acc	MAA	M (SD)	0.224 (0.079)	0.221 (0.079)	0.219 (0.083)	0.223 (0.079)	0.224 (0.084)	0.098 (0.032)	0.101 (0.032)	0.098 (0.031)	0.102 (0.034)	0.101 (0.035)	0.263 (0.069)	0.262 (0.068)	0.258 (0.073)	0.264 (0.067)	0.266 (0.073)
		MFA	M (SD)	1.380 (0.344)	1.402 (0.304)	1.362 (0.320)	1.389 (0.310)	1.372 (0.330)	1.562 (0.558)	1.540 (0.587)	1.516 (0.547)	1.567 (0.621)	1.532 (0.580)	1.345 (0.254)	1.358 (0.254)	1.321 (0.251)	1.346 (0.253)	1.323 (0.266)
		NPL	M (SD)	1.818 (0.377)	1.837 (0.414)	1.759 (0.429)	1.838 (0.434)	1.824 (0.505)	0.884 (0.228)	0.886 (0.230)	0.855 (0.233)	0.911 (0.276)	0.882 (0.235)	2.163 (0.446)	2.181 (0.480)	2.091 (0.496)	2.195 (0.518)	2.167 (0.564)
		P2P	M (SD)	**0.461 (0.081)**	**0.462**^**#**^**(0.095)**	**0.433**^**#**^**(0.083)**	**0.446 (0.083)**	**0.458 (0.105)**	0.284 (0.061)	0.291 (0.056)	0.274 (0.054)	0.273 (0.058)	0.288 (0.072)					
		RMS	M (SD)	**0.156**^**ˆ**^**(0.052)**	**0.156**^**#**^**(0.054)**	**0.152**^**#ˆ**^**(0.053)**	**0.153 (0.053)**	**0.155 (0.056)**	0.068 (0.019)	0.071 (0.019)	0.068 (0.019)	0.069 (0.020)	0.070 (0.022)	**0.173 (0.047)**	**0.174**^**#**^**(0.047)**	**0.169**^**#**^**(0.046)**	**0.171 (0.046)**	**0.173 (0.049)**
	Acc_X_	MAA	M (SD)	**0.106 (0.019)**	**0.107**^**#**^**(0.021)**	**0.103**^**#**^**(0.020)**	**0.104 (0.021)**	**0.105 (0.023)**	0.042 (0.013)	0.044 (0.014)	0.042 (0.014)	0.044 (0.015)	0.044 (0.014)	**0.122 (0.019)**	**0.124**^**#**^**(0.020)**	**0.119**^**#**^**(0.019)**	**0.121 (0.021)**	**0.121 (0.021)**
		MFA	M (SD)	1.840 (0.155)	1.845 (0.161)	1.804 (0.156)	1.829 (0.179)	1.813 (0.187)	2.277 (0.620)	2.184 (0.619)	2.167 (0.582)	2.197 (0.650)	2.160 (0.619)	1.876 (0.224)	1.858 (0.231)	1.823 (0.219)	1.855 (0.271)	1.831 (0.255)
		NPL	M (SD)	**1.240 (0.289)**	**1.253**^**#**^**(0.308)**	**1.178**^**#**^**(0.293)**	**1.206 (0.323)**	**1.207 (0.342)**	0.573 (0.170)	0.574 (0.178)	0.544 (0.164)	0.578 (0.207)	0.558 (0.176)	**1.455 (0.341)**	**1.465**^**#**^**(0.361)**	**1.379**^**#**^**(0.341)**	**1.425 (0.390)**	**1.414 (0.387)**
		P2P	M (SD)	0.447 (0.086)	0.455 (0.096)	0.430 (0.082)	0.435 (0.089)	0.446 (0.110)	0.234 (0.050)	0.232 (0.052)	0.222 (0.049)	0.228 (0.059)	0.232 (0.051)					
		RMS	M (SD)	**0.122 (0.022)**	**0.123**^**#**^**(0.024)**	**0.118**^**#**^**(0.023)**	**0.119 (0.025)**	**0.120 (0.027)**	0.051 (0.014)	0.053 (0.015)	0.050 (0.015)	0.052 (0.016)	0.052 (0.015)	**0.133 (0.021)**	**0.135**^**#**^**(0.022)**	**0.129**^**#**^**(0.022)**	**0.131 (0.023)**	**0.132 (0.025)**

#: Significant pairwise comparison between DT2 and DT3 (p < 0.05); ˆ: significant pairwisw comparison between DT1 and DT3 (p = 0.027); Bold cells indicates significant effect of dual-task conditions (p < 0.05)RMS: root mean square; P2P: Peak to Peak acceleration; NPL: normalized path length; MFA: mean frequency of acceleration; MAA: mean absolute acceleration; ML: mediolateral direction; AP: anterior-posterior direction; 2D: combined direction; Acc: filtered acceleration; Acc_X_: mean referenced filtered acceleration; 2Hz: 2 Hz cutoff frequency of a low pass filter; 3.5Hz: 3.5 Hz cutoff frequency of a low pass filter; DT1: serial subtractions by 7's; DT2: naming words that start with a certain letter; DT3: reverse reciting of the months; DT4: normal conversation; DT5: reverse spelling; M: mean; SD: standard deviation.

Four one-way repeated-measures MANOVA were performed to evaluate the effect of dual-task conditions on sway data calculated using the 56 calculation methods. Mauchly's test indicated that the assumption of sphericity had been violated in some dependent variables, in those cases the degrees of freedom were corrected using Greenhouse-Geisser estimates of sphericity. Results showed that 10 of the 56 calculation methods were significantly affected by the dual-task conditions (Table 2).

**Table 2:** Effects of dual-task conditions on sway.

Normalization		AccX							Acc						
Filter	Measures	Mauchly's			Univariate Test				Mauchly's			Univariate Test			
		W	P value	df	f	df	P value	Error df	W	P value	df	F	df	P value	Error df
2 Hz	NPL_AP	0.626	0.278	9	2.327	4	0.061	100	0.666	0.392	9	0.793	4	0.532	100
	NPL_ML	0.515	0.077	9	1.425	4	0.231	100	0.500	0.063	9	1.991	4	0.102	100
	NPL_2D	0.628	0.283	9	2.483	4	0.048∗	100	0.676	0.424	9	1.159	4	0.334	100
	RMS_AP	0.446	0.026^#^	9	*0.887*	*3.21*	*0.457*	*80.12*	0.483	0.049^#^	9	*1.175*	*3.11*	*0.325*	*77.72*
	RMS_ML	0.501	0.064	9	1.715	4	0.153	100	0.258	0.000^#^	9	*1.047*	*2.50*	*0.369*	*62.46*
	RMS_2D	0.559	0.138	9	1.504	4	0.207	100	0.368	0.005^#^	9	*1.455*	*2.68*	*0.237*	*67.05*
	MAA_AP	0.450	0.028^#^	9	*0.894*	*3.20*	*0.454*	*80.10*	0.542	0.112	9	0.631	4	0.642	100
	MAA_ML	0.525	0.089	9	1.746	4	0.146	100	0.285	0.001^#^	9	*0.497*	*2.47*	*0.650*	*61.72*
	MAA_2D	0.568	0.153	9	1.731	4	0.149	100	0.484	0.049^#^	9	*0.968*	*3.10*	*0.414*	*77.44*
	P2P_AP	0.588	0.192	9	0.423	4	0.792	100	0.720	0.565	9	0.842	4	0.502	100
	P2P_ML	0.445	0.026^#^	9	*0.693*	*2.92*	*0.556*	*73.00*	0.755	0.682	9	0.478	4	0.752	100
	MFA_AP	0.583	0.182	9	2.233	4	0.071	100	0.537	0.104	9	0.601	4	0.663	100
	MFA_ML	0.663	0.382	9	1.830	4	0.129	100	0.302	0.001^#^	9	*1.324*	*2.40*	*0.275*	*60.08*
	MFA_2D	0.631	0.293	9	1.863	4	0.123	100	0.300	0.001^#^	9	*0.510*	*2.33*	*0.631*	*58.23*
3.5 Hz	NPL_AP	0.500	0.063	9	2.739	4	0.033∗	100	0.589	0.193	9	1.429	4	0.230	100
	NPL_ML	0.435	0.022^#^	9	*1.062*	*3.21*	*0.373*	*80.34*	0.584	0.182	9	1.313	4	0.270	100
	NPL_2D	0.553	0.127	9	2.580	4	0.042∗	100	0.485	0.050^#^	9	*1.653*	*3.05*	*0.183*	*76.36*
	RMS_AP	0.542	0.112	9	2.701	4	0.035∗	100	0.538	0.105	9	2.538	4	0.045∗	100
	RMS_ML	0.522	0.086	9	1.633	4	0.172	100	0.316	0.001^#^	9	*1.475*	*2.65*	*0.232*	*66.22*
	RMS_2D	0.575	0.166	9	2.829	4	0.029∗	100	0.672	0.411	9	2.807	4	0.030∗	100
	MAA_AP	0.589	0.193	9	2.718	4	0.034∗	100	0.617	0.256	9	0.844	4	0.500	100
	MAA_ML	0.491	0.055	9	1.992	4	0.101	100	0.222	0.000^#^	9	*1.183*	*2.58*	*0.320*	*64.52*
	MAA_2D	0.600	0.216	9	2.982	4	0.023∗	100	0.563	0.143	9	1.458	4	0.221	100
	P2P_AP	0.555	0.132	9	2.260	4	0.068	100	0.603	0.224	9	2.842	4	0.028∗	100
	P2P_ML	0.503	0.065	9	1.274	4	0.285	100	0.656	0.363	9	1.199	4	0.316	100
	MFA_AP	0.587	0.190	9	1.773	4	0.140	100	0.516	0.079	9	0.904	4	0.464	100
	MFA_ML	0.679	0.433	9	1.484	4	0.213	100	0.418	0.016^#^	9	*0.321*	*2.95*	*0.807*	*73.86*
	MFA_2D	0.663	0.382	9	2.079	4	0.089	100	0.335	0.002^#^	9	*0.922*	*2.69*	*0.427*	*67.34*

^#^: Sphericity Assumption has been violated; ∗: significant effect of dual-task conditions; *Italicized* numbers: Greenhouse-Geisser correction.

RMS: root mean square; P2P: Peak to Peak acceleration; NPL: normalized path length; MFA: mean frequency of acceleration; MAA: mean absolute acceleration; ML: mediolateral direction; AP: anterior-posterior direction; 2D: combined direction; Acc: filtered acceleration; Acc_X_: mean referenced filtered acceleration; 2Hz: 2 Hz cutoff frequency of a low pass filter; 3.5Hz: 3.5 Hz cutoff frequency of a low pass filter; DT1: serial subtractions by 7's; DT2: naming words that start with a certain letter; DT3: reverse reciting of the months; DT4: normal conversation; DT5: reverse spelling.

The none mean-referenced 2 Hz low-pass filtered sway did not capture significant effects of dual-tasks. However, the mean-referenced 2 Hz low-pass filter found a significant effect of dual-task conditions on the NPL of sway in the 2D direction, F(4, 100) = 2.483, p = 0.048. Post-hoc pairwise comparisons with a Bonferroni adjustment indicated that task of naming words that start with a certain letter had higher NPL in 2D than the reverse reciting of the months task, (*p* = 0.006).

The 3.5 Hz low-pass filtered sway captured significant effects of dual-tasks in both mean-referenced and none mean-referenced sway. The mean-referenced 3.5 Hz low-pass filtered sway captured significant effect of dual-task conditions on 3 of the 5 computational methods of sway (MAA, NPL, and RMS) on 2 of the 3 directions of sway (AP and 2D). The none mean-referenced 3.5 Hz low-pass filtered sway captured significant effects of dual-task conditions RMS of sway in AP and 2D directions. Furthermore, the P2P computational method captured a significant effect of dual-task conditions in the AP direction. Post-hoc pairwise comparisons with Bonferroni adjustments indicated that in all of the 9 cases, of significant effects of dual-task conditions on the 3.5 Hz low-pass filtered sway, the task of naming words that start with a certain letter had higher sway than the reverse reciting of the months task, (p < 0.05). Furthermore, a post-hoc pairwise comparison with Bonferroni adjustment indicated that P2P of sway in the AP direction was higher during the serial subtractions by 7's task than the reverse reciting of the months task, (p = 0.027). See Table 1 for more details.

### 3.1 Direction of sway

Significant changes in sway were observed in the AP and 2D directions under the different dual-task conditions, with no significant changes detected in the ML direction. Both the AP and 2D directions had 5 sway measures capturing significant effects of dual-tasks, while none of the sway measures in the ML direction captured these effects.

### 3.2 Frequency cutoff

The 3.5 Hz frequency cutoff consistently captured changes in sway magnitude, with nine measures reflecting significant differences between the dual-task conditions. Conversely, the 2 Hz frequency cutoff captured significant effects in only one measure.

### 3.3 Mean reference

Using the non-mean-referenced data (Acc), three sway measures captured significant effects of dual-tasks. While seven measures of sway using the mean-referenced data (Acc_x_) captured significant dual-tasks effects.

### 3.4 Computation method

The RMS, NPL, MAA, and P2P captured significant effects of dual-tasks on sway, whereas the MFA did not capture dual-tasks effects in any of the 12 combinations of sway data.

The RMS captured significant dual-task effects in the 3.5Hz low-pass filtered Acc and Acc_X_ data for the AP and 2D directions, but not the ML direction. The RMS for all 2Hz low-pass filtered data did not capture dual-task effects.

The NPL captured significant dual-task effects in the 3.5Hz low-pass filtered Acc_X_ data for the AP and 2D directions, as well as in the 2Hz Acc_X_ data for the 2D direction. NPL did not capture dual-task effects in the Acc data or the ML direction.

The MAA captured significant dual-task effects in the 3.5Hz low-pass filtered Acc_X_ data for the AP and 2D directions. It did not capture dual-task effects in any of the 2Hz low-pass filtered data, Acc data, or ML direction.

The P2P was computed in the AP and ML directions only. It captured significant dual-task effects in the 3.5Hz low-pass filtered Acc data for the AP direction. However, P2P did not capture dual-task effects in any of the 2Hz low-pass filtered data, Acc_X_ data, or ML direction.

## 4 Discussion

The findings provided important insights into the effects of dual-task conditions on walking sway and the utility of accelerometer-based metrics for balance assessment. The main findings were: 1) All of the computation methods, except for the MFA, were able to capture significant change in walking sway between the different dual-tasks. 2) The AP and 2D sway were able to capture change in walking sway during the different dual-tasks while the ML direction did not. 3) The 2Hz low-pass filter was inferior to the 3.5 low-pass filter in capturing change in walking sway during the different dual-tasks. 4) Both Acc and Acc_X_ were able to capture significant change in walking sway between the different dual-tasks.

The importance of using the Acc vs Acc_X_ depended on the computational method that is used. Using Acc_X_, the mean referenced sway, found to be necessary for the NPL and the MAA measures to capture change in walking sway during the different dual-tasks. While using Acc, the sway data without mean referencing, was necessary for the P2P measure to capture change in walking sway during the different dual-tasks. We found that the RMS was able to capture change in walking sway during the different dual-tasks using both Acc and Acc_X_. The RMS finding was expected as the RMS method of computation includes mean referencing the data. The RMS method's ability to detect changes across both mean-referenced and non-mean-referenced data highlights its robustness as a general metric for dual-task assessments.

Four computation methods (RMS, NPL, MAA, and P2P) captured significant dual-task effects on sway, whereas MFA did not. Martinez-Mendez et al. (2012) reported that RMS effectively differentiated between younger and older adults in postural sway analysis using accelerometers, reinforcing its value as a sensitive measure [25]. Similarly, Whitney et al. (2011) compared accelerometry and center of pressure metrics, concluding that accelerometer-derived RMS is comparable to traditional force plate measures in evaluating postural control [23].

The AP and 2D sway were able to capture changes in walking sway during various dual-task conditions, whereas the ML direction did not exhibit significant variations. The discrepancy in capturing the dual-tasks effect between the AP and ML directions aligns with results from a previous study by Tramontano et al., 2016, which demonstrate increased ML acceleration and decreased AP acceleration during dual-tasking [31]. This supports the idea that AP and ML sway respond differently to cognitive dual-tasking, emphasizing the need to consider both axes in assessing sway. However, Eagle et al. (2021), who focused on standing balance, found that both AP and ML sway were significantly greater in concussed adolescents, suggesting that task type and participant characteristics may modulate directional sway responses [7]. Sebastia-Amat et al. (2023) further demonstrated that AP and ML sway may respond differently depending on the nature of the task. In Parkinson's disease, ML sway, unlike AP sway, increases when visual feedback is removed [21]. This distinction underscores the importance of task-specific analyses when assessing balance metrics.

## 5 Conclusion

To capture changes in walking sway during dual-tasking, our results suggest computing RMS, NPL, or MAA using the mean referenced AP and 2D acceleration data filtered with a 3.5Hz low-pass filter. Furthermore, the P2P was found to be effective to capture changes in walking sway during dual-tasking when computed using the non-mean referenced acceleration data in the AP direction.

## 6 Limitations

This study has several limitations that should be considered when interpreting the findings, including the healthy participant group, the differences in dual-task complexity, the specific computational methods used, and the small sample size.

The cognitive tasks used in the dual-task paradigm varied in difficulty, which may have influenced participants' performance. The study did not measure or control for these differences in cognitive load, which could have affected the results. The study utilized specific filtering cutoffs and computational methods. While these choices were based on prior research, they might not cover all potentially useful methods or filters for sway analysis.

The study included only healthy young adults with no balance impairments or neurological conditions. This limits how well the findings apply to groups with more significant balance challenges, such as older adults or individuals with neurological disorders. Lastly, the small sample size of 26 participants likely reduced the statistical power of the study, limiting its ability to detect subtle differences or interactions. This should be considered when interpreting the non-significant results reported.

## Ethics approval and consent to participate

All methods were carried out in accordance with relevant guidelines and regulations. This study was reviewed and approved by the regional research ethics committee, Registered at National Committee of Bio & Med. Ethics (NCBE) at Qassim Province with the approval number: H-04-Q-001, dated December 27, 2020.

All participants provided written informed consent to participate in the study and for their data to be published.

## Availability of data and materials

The datasets used and/or analyzed during the current study are available from the corresponding author on reasonable request.

## Declaration of competing interest

The authors declare the following financial interests/personal relationships which may be considered as potential competing interests: Abdulaziz A. Alkathiry reports article publishing charges was provided by Majmaah University. If there are other authors, they declare that they have no known competing financial interests or personal relationships that could have appeared to influence the work reported in this paper.
